# Effects of Climatic Conditions on the Lying Behavior of a Group of Primiparous Dairy Cows

**DOI:** 10.3390/ani9110869

**Published:** 2019-10-26

**Authors:** Emanuela Tullo, Gabriele Mattachini, Elisabetta Riva, Alberto Finzi, Giorgio Provolo, Marcella Guarino

**Affiliations:** 1 Department of Environmental Science and Policy, Università degli Studi di Milano, 20133 Milano, Italy; marcella.guarino@unimi.it; 2 Department of Agricultural and Environmental Sciences, Università degli Studi di Milano, 20133 Milano, Italy; mxlelex@libero.it (G.M.); elisabetta.riva@unimi.it (E.R.); alberto.finzi@unimi.it (A.F.); giorgio.provolo@unimi.it (G.P.)

**Keywords:** lying behavior, climatic conditions, temperature-humidity index (THI), dairy cows, accelerometers, prediction model

## Abstract

**Simple Summary:**

Dairy cow welfare has become a significant topic in recent years. Lying (down) behavior is considered a useful indicator for dairy cow health, welfare, reproductive and productive status. The study evaluated the interaction of climatic conditions on the lying behavior of a group of dairy cows. The developed model seems helpful to identify and predict this important indicator of the welfare of the herd. The prediction model developed, with automatic monitoring of cow behavior, could be a valid early warning system to identify any deviation from the expected behavior, and could be also used to evaluate the goodness of management and to evaluate the heat stress mitigation strategy.

**Abstract:**

Currently, lying behavior can be assessed using continuous observations from sensors (e.g., accelerometers). The analysis of digital data deriving from accelerometers is an effective tool for studying livestock behaviors. Despite the large interest in the lying behavior of dairy cows, no reference was found in literature regarding the prediction of lying behavior as a function of the interaction of environmental parameters. The present study aimed to evaluate the influence of climatic conditions (temperature-humidity index, solar radiation, air velocity and rainfalls) on the lying behavior of a group of primiparous dairy cows, using data from accelerometers, and develop a prediction model to identify and predict the lying behavior of dairy cows as a function of the effects of environmental conditions. Results from the. GLM Procedure (SAS) showed that the model was highly significant (*p* < 0.001) and the r^2^ was 0.84. All of the effects in the model resulted in being highly significant (*p* < 0.001). This model, if validated properly, could be a valid early warning system to identify any deviation from the expected behavior, and to assess the effectiveness of thermal stress mitigation strategies.

## 1. Introduction

Dairy cow welfare has become a significant topic in recent years. Time spent lying down, the frequency and the duration of lying bouts, are considered to be useful indicators for dairy cow health, welfare, reproductive and productive status [1,2]. Dairy cows spend between 9 and 14 h/d lying down, also in barns with Automated Milking Systems (AMS), and they prioritize resting over other behaviors [2,3], even on feeding behavior [4].

Over the course of the day, this time spent lying down varies, with a peak before morning milking, and in the middle of the day between the two daily milkings [5]. In AMS barns, regardless of the type of traffic system, diurnal patterns of feeding and lying behavior persist, with more cows lying down overnight [6].

Lying down and resting behaviors are important for the cow, and often, better welfare is associated with longer lying periods [7]. Changes in lying behavior can be caused by diseases, housing conditions, stocking density, temperature, barn design and several other factors [1,8,9]. However, when lying time become too long, it may be a symptom of pathology or the manifestation of a problem such as lameness or metritis [1,2,8,10], while shorter periods may be associated with severe mastitis or ketosis [11,12]. For these reasons, monitoring this behavior plays an important role in herd management.

Currently, lying behavior can be assessed using continuous observations from video recordings or data from sensors (e.g., Accelerometers) [8,13,14]. The analysis of digital images deriving from video-recordings is an effective tool for studying livestock behaviors, although it does have some drawbacks, such as the large amount of time used to manually check the files, and the possible mismatch in the interpretation among observers [15].

Likewise, accelerometers and bio-logger have the great potential of providing large amounts of behavioral data for days or months, and their use is spreading compared to video analysis due to reduced size, weight, low cost and embedded algorithms that provide valuable information regarding individual behaviors and dynamic processes within the herd [13]. Use of these devices to measure lying behavior has become increasingly common, due to the high level of accuracy and sensitivity [16]. Information on behaviors deriving from sensors can be used as a clear welfare indicator [17], and especially information regarding cow lying behavior can indicate the level of welfare of the considered herd.

Lying behavior in free stall barns is affected by barn design and management factors [18,19], social relationships between animals [5], milk production [20] and the health status of cows [10,21]. Lying behavior is also influenced by the stage of lactation [22]. For instance, lying time and lying bout duration increase with increasing days in milk (DIM) [23]. Particularly, cows in early lactation spend more time eating and less time lying than cows in late lactation [22]. Moreover, physical activity in AMS farms, and consequently lying behavior, can be influenced by the length of daylight, the season, or by geographical factors such as the latitude at which the barn is located [24].

Furthermore, the lying time can be used as a measure of a cow’s welfare, also in the case of heat stress [25], that seriously affects feed intake, cow body temperature, maintenance requirements and metabolic processes, feed efficiency, milk yield, reproductive efficiency, cow behavior and disease incidence [26,27].

In particular, reduced milk productivity and reproductive disorders, such as silent estrus and calving difficulty, can seriously affect the economic balance of the farm [28].

Heat stress is an important threat to cattle breeding, especially in the Mediterranean basin [29], where the combination of high temperatures and high humidity can result in harsh conditions for dairy cows. This situation can be exacerbated by air velocity and the intensity of solar radiation, particularly in unshaded areas of a barn [25]. Dairy cows use several physiological strategies to cope with heat stress, including increased respiration rate, panting and sweating, and reduced milk yield. Additionally, dairy cows modify their behavior in terms of drinking and feed intake (e.g., increased water intake and shifting feeding times to cooler periods during the day), increased standing time and shade seeking, and decreased activity and movement [30].

Experiencing uncomfortable thermal conditions (due to the combination of high temperature and humidity, solar radiation and air velocity) affects the capacity of cattle to dissipate heat, and leads to an increase in body temperature over the physiological limits [31].

Several authors evaluated the effects of heat stress in this species using the temperature-humidity index (THI), an index that combines the simultaneous effect of temperature and humidity, finding a good relationship between this index and the welfare status of cows [25,29,32,33].

Recently, a predictive model for physiological responses, which incorporated air temperature, relative humidity, air velocity and solar radiation and their interactions, was developed and the results were mildly correlated with skin and body temperature and respiration rate [34].

However, despite the large interest in the lying behavior of dairy cows, no reference was found in literature regarding the description of lying behavior as a function of the interaction of environmental parameters.

The present study aimed to evaluate the influence of climatic conditions (THI, solar radiation, air velocity and rainfalls) on the lying behavior of a group of primiparous dairy cows, using data from accelerometers and developing a model to describe the lying behavior of dairy cows as a function of the effects of environmental conditions.

## 2. Materials and Methods

### 2.1. Animals and Housing

**Figure 1:**
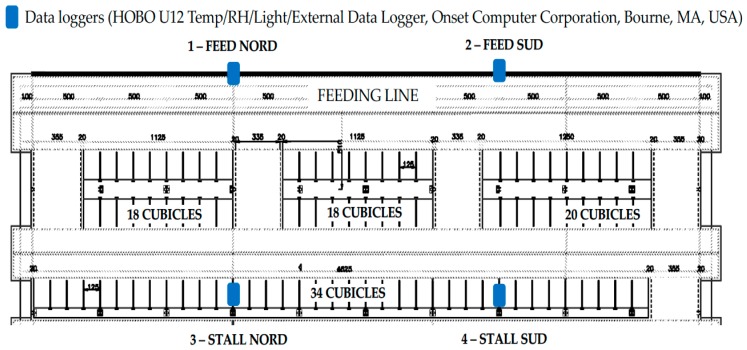
Layout of the barn and location of the monitoring equipment during the trial.

Data were collected at the experimental farm A. Menozzi (Landriano, Italy; 45°19′16.5″ N, 9°15′56.4″ E) of the University of Milan, between October 2013 and August of 2014. Dairy Cows were housed in a free-stall pen in a loose-housing layout with a total of 90 cubicles having rubber mats and 60 feeding places (Figure 1).

In the study a total of 21 primiparous Holstein dairy cows (age at calving = 2.30 ± 0.221 years, average daily milk yield 26.51 ± 7.38 kg) were monitored. For each cow, the monitoring period started from the first days after calving and lasted around 150 days. Parturition period ranged from 3 September 2013 to 27 May 2014. The primiparous group belonged to the same feeding group, the proportion between the number of cows and the number of pens and feeding places was 1.5 and 0.9, respectively.

A total mixed ration (TMR) was delivered once daily beginning at approximately 10:00. Cows had ad libitum access to six water troughs and were fed a TMR consisting of 33.6% maize silage, 18.5% high moisture corn 12.5% concentrate, 8.4% soybean meal, 6.7% maize meal, 6.2% alfalfa hay, 5.4% cotton seeds, 4.7% grass hay, 2.6% molasses and 1.6% mineral supplement by dry weight. Cows were milked two times daily at approximately 08:30 and 21:00. Stalls were cleaned manually once daily at approximately 09:00.

Feed bunk had sprinkler nozzles spaced 1 m apart; these were mounted about 2 m high and angled to avoid wetting the feed, creating a spray radius extending approximately 1.5 m from the feed bunk. The cooling system has been activated when the temperature-humidity index (THI) reached a value of 68.

### 2.2. Behavioral Data

Lying behavior patterns of 21 cows were automatically recorded for 342 days using HOBO Pendant G Data Loggers (Onset Computer Corporation, Pocasset, MA, USA). In the first week cows were equipped with an individual HOBO Data Logger, and their activities recorded continuously for 150 days. These devices measured leg orientation at 1-min intervals, and allowed all the standing and lying behavior data to be collected electronically [16]. The devices were installed to the lateral side of the right hind leg of each cow by using plastic tough leg bands in a position such that the *x*-axis of the data logger was perpendicular to the ground. The degree of vertical tilt of the *x*- and *z*-axis was used to determine the standing and lying behavior of the animal [16]. Data collected by the data loggers were used to calculate standing and lying times (h/d) for each cow and each day during trial monitoring. Due to the limited memory space of these devices, every three weeks the data where downloaded from the loggers during morning milking.

Data collection for this study was performed at a commercial farm, and all the monitoring actions and procedures did not affect the behavior of the cows, and did not change the comfort or welfare of the animals monitored. All the used sensors are widely used in dairy husbandry, since they are not invasive for cows.

### 2.3. Environmental Data

Four data loggers were used to measure the air temperature and relative humidity continuously during the trial duration (HOBO U12 Temp/RH/Light/External Data Logger, Onset Computer Corporation, Bourne, MA, USA). The data loggers were placed in four separate locations at a height of about 2 m above the floor (Figure 1). The recording interval for microclimatic data was set at 30 min. temperature-humidity indices (THI) were calculated using the following calculation:

(1) $$\mathrm{THI}\;=\;\left\{1.8\times T-\;\left[\frac{1-RH}{100}\right]\times \left(T-14.3\right)+32\right\}$$

where *T* is the temperature in °C, and *RH* is the relative humidity [35].

An average THI was determined from the calculated THI for each position in the barn.

A weather station located in the farm (Landriano, Cascina Marianna, Italy; 45°19′14.1″ N 9°16′01.8″ E), and part of the regional meteorological network (https://www.dati.lombardia.it/Ambiente/Stazioni-Meteorologiche/nf78-nj6b) also provided information relative to temperature (°C), relative humidity (%), global radiation (W/m^2^), precipitation (mm) and wind speed (m/s).

### 2.4. Data Processing and Statistical Analysis

Missing data, deriving principally from cows culled from the production due to health problems or from the malfunctioning of the accelerometers, were not included in the model. Malfunctioning occurred, since the device used in the trial were used for longer periods without a shielding suitable for the harsh environment of a dairy cow’s barn.

Environmental data collected in four locations were averaged for each day, and prior to the analysis, these were cleaned from outliers in order to have more homogeneous distributions of data.

At the end of the editing process, only the 2% of the original dataset was removed and the final dataset included accelerometers’ data for 19 cows, and on average, the individual recording period lasted for 138 days.

To evaluate the influence of climatic conditions on lying behavior, a Generalized Linear Model (GLM) was applied (Proc GLM, SAS, Cary, NC, USA). In the model, the mean daily lying time (LY) was modeled as a function of the week of the year and of the interaction of THI, solar radiation (RAD), air velocity (WIND) and precipitation (RAIN) (LY = WEEK + (THI × RAD × WIND × RAIN)).

Despite the presence of THI, that takes in account the air moisture, precipitation was included in the model to evaluate the effect of this parameter on lying behavior. Indeed, as reported by Bohmanova et al. [36] heat stress is caused by a combination of environmental factors (temperature, relative humidity, solar radiation, air movement and precipitation) but often data on the solar radiation received by the animal, wind speed and rainfall are not publicly available.

In this study all these data were available and were all included in the model to make the model more robust. Furthermore, all the variables in the data set were grouped into classes, to avoid the inhomogeneity of the sample. The classes included in the model were: The week of the year (WEEK, 49 classes), the THI (five classes), the solar radiation (RAD, four classes), the air velocity (WIND, four classes) and precipitation (RAIN, two classes). The division in classes was performed according the classification of winds for the WIND parameter, and according the Notes on the Temperature-Humidity Index (NOAA, National Weather Service) for the THI parameter. The RAD was classified according the distribution of data, and the precipitation parameter was divided in the presence or absence of rain (Table 1).

This model allows us to describe the expected mean lying time based on the combination of each class of each effect with the other classes of the other effects. As an example, it allows us to estimate the mean daily lying time when the THI is in the <50 class combined with a RAD < 40 W/m^2^, WIND < 1 m/s and no rain, in a specific week of the year.

At the end of the analysis, predicted values resulting from the GLM procedure were then compared with observed values with the PROC CORR (SAS, Cary, NC, USA).

## 3. Results

### Behavioral and Environmental Data

**Figure 2:**
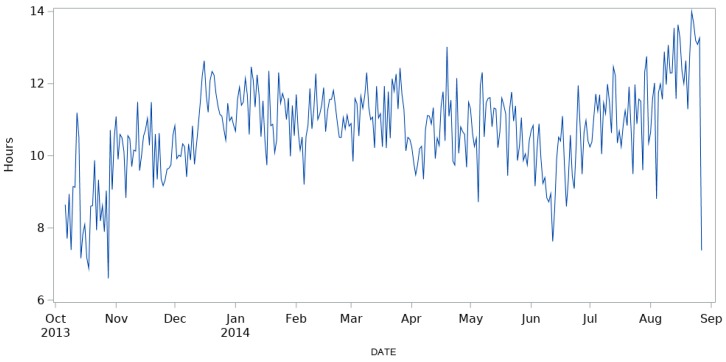
Mean daily values of the lying time of the monitored herd during the trial.

Mean daily lying time reported in Figure 2 showed the high variability in the time spent lying by the cows. Indeed, time varied from 6.6 to 14 h, and varied also according to the month.

**Figure 3:**
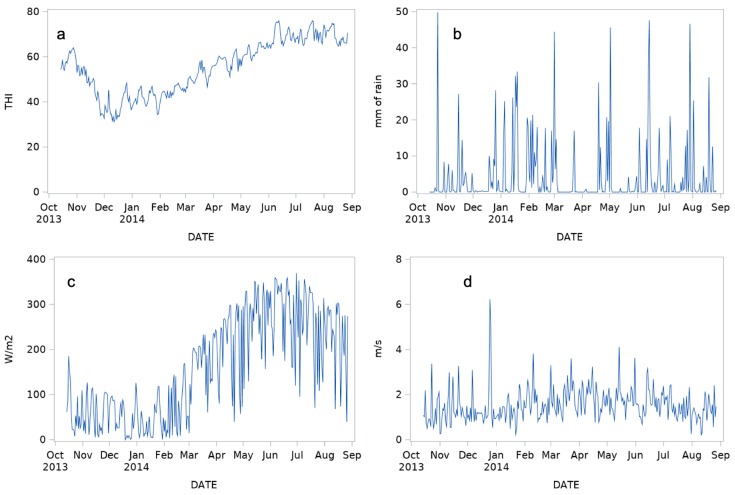
Daily variation of the Temperature Humidity Index (THI) (**a**), precipitation (RAIN) (**b**), solar radiation (RAD) (**c**) and air velocity (WIND) (**d**) during the trial.

Daily variation of the Temperature Humidity Index (THI), precipitation (RAIN), solar radiation (RAD) and air velocity (WIND) during the trial is reported in Figure 3.

**Figure 4:**
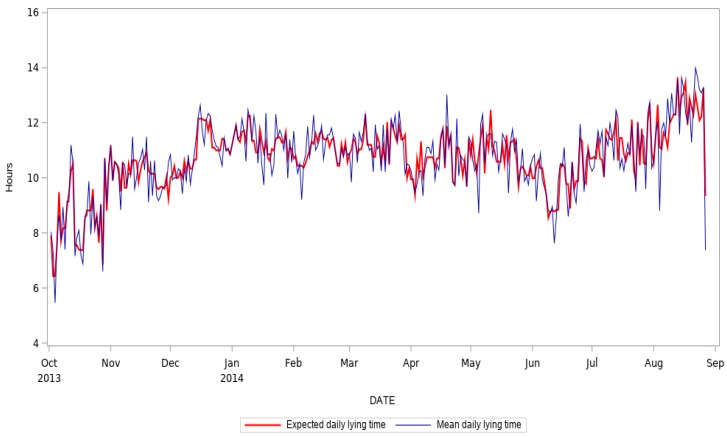
Expected daily lying time (red line) and observed mean daily lying time (blue line).

Results from the PROC GLM showed that the model was highly significant (*p* < 0.001) and the r^2^ was 0.84. All of the effects in the model resulted in being highly significant (*p* < 0.001). In Figure 4, the mean daily expected lying time (in hours—red line), compared to the observed mean daily lying time (in hours—blue line).

**Figure 5:**
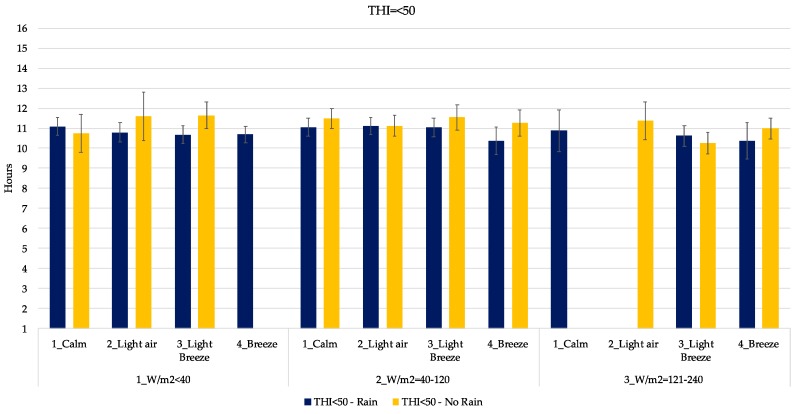
Estimation of the mean lying time when THI is below 50. Blue bars indicate rainy days. *X*-axis gives the indication of WIND and RAD expressed as W/m^2^. Error bars represent the standard error.

**Figure 6:**
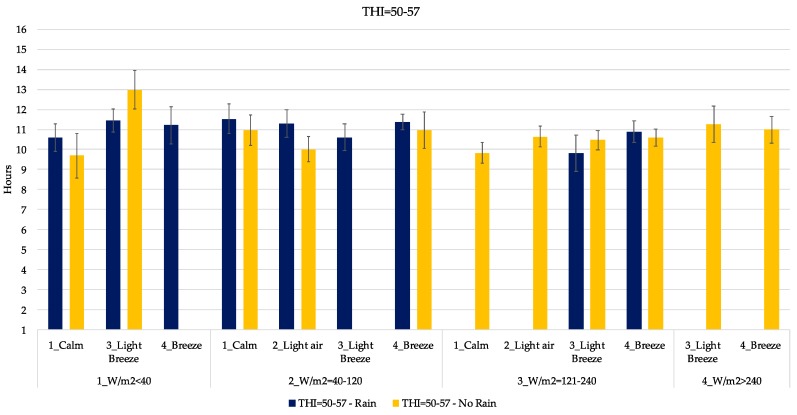
Estimation of the lying time when THI is included in the range 50–57. Blue bars indicate rainy days. *X*-axis gives the indication of WIND and RAD expressed as W/m^2^. Error bars represent the standard error.

**Figure 7:**
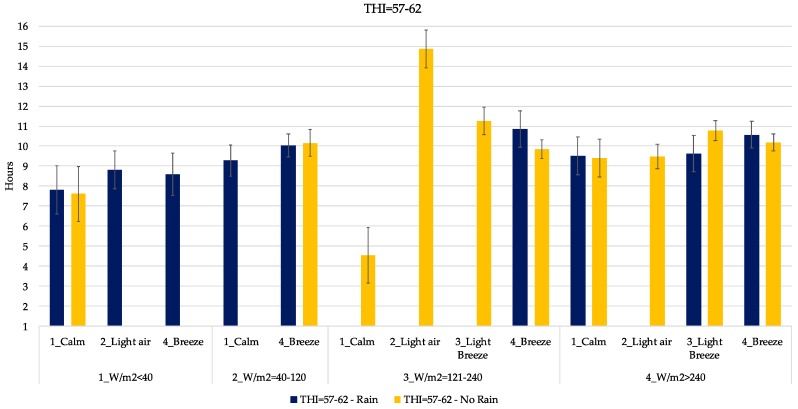
Estimation of the lying time when the THI is included in the range 57–62. Blue bars indicate rainy days. *X*-axis gives the indication of WIND and RAD expressed as W/m^2^. Error bars represent the standard error.

**Figure 8:**
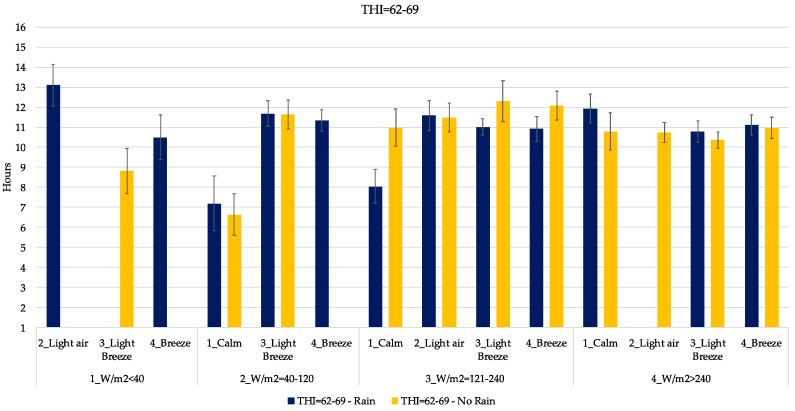
Estimation of the lying time when THI is included in the range 62–69. Blue bars indicate rainy days. *X*-axis gives the indication of WIND and RAD expressed as W/m^2^. Error bars represent the standard error.

**Figure 9:**
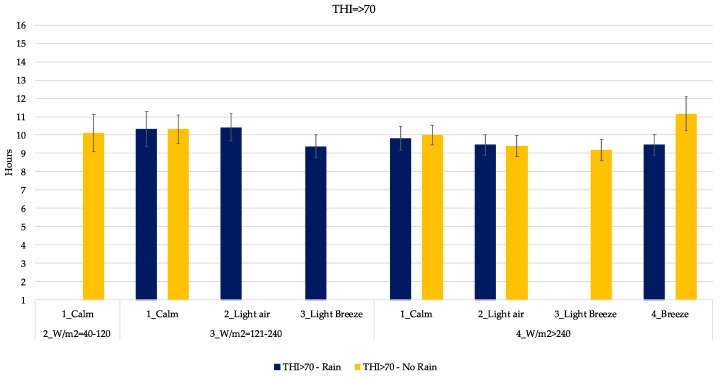
Least Square Means estimation of the lying time when THI is above 70. Blue bars indicate rainy days. *X*-axis gives the indication of WIND and RAD expressed as W/m^2^. Error bars represent the standard error.

In Figure 5, Figure 6, Figure 7, Figure 8 and Figure 9, the mean daily lying time estimation of the interaction among THI, RAD, WIND and RAIN. In each graph, clustered by THI class, there is the expected mean lying time in the different climatic condition found during the study. Indeed, each graph is furtherly subdivided in the presence and absence of RAIN, power of RAD and WIND.

For example, in Figure 5 the expected mean lying time is 11 h when the climatic conditions are the following: THI < 50, WIND = calm, RAD < 40 W/m^2^ and RAIN = yes. Missing bars represent missing events, given as an example: the data set did not include a combination of climatic conditions with THI < 50, WIND = breeze, RAD < 40 W/m^2^ and RAIN = no (Figure 5). Thus, the duration of the mean lying time in these conditions could not be estimated.

From Figure 5 it is possible to see how the expected mean lying time is almost always included in the 10–11.7 h range when the THI is below 50. At this value of THI, it seems that the difference between rainy and clear days is not so relevant, even if there is some wind.

Regarding THI between 50 and 57, that corresponds to a temperature of 8–13 °C (Figure 6), there is a higher variability in the lying behavior. Indeed, there is difference between rainy and clear days (especially when the RAD is lower than 120 W/m^2^). During rainy days cows tend to spend more time lying respect to clear days (11.4 h vs 9.2 h in average). In this range of THI the lying time range is 9.6–11.7 h, slightly lower than the previous class (THI > 50). During sunny days (RAD above 121 W/m^2^), dairy cows increased the time spent lying as the WIND increased, reaching and exceeding the 11 h in the cubicle.

With THI values included in the range of 57–62 (between 13 and 19 °C, Figure 7), the trend for time spent down varied a lot.

The general tendency for the lying time is much lower than the previous classes, and, except for a couple of values, the expected lying time is lower than 10 h. What is worthy to note is that at the same RAD, what affects the behavior more is the WIND. The highest value (more than 14 h) is expected when the RAD is in a sunny day, between 121 and 240 W/m^2^, and with light air (1.3–1.8 m/s). Also, in this case (THI = 57–62) the time spent lying increased with increasing RAD and WIND.

When the THI increased between 62 and 69, (temperatures between 19 and 24 °C), the expected lying time ranged from 6.5 to 13 h (Figure 8). The maximum was reached during a rainy day with light air, while the minimum is expected in a calm, sunny day. Above 121 W/m^2^ the lying time exceeded 10 h, increasing with the increase of WIND.

Over a value of 70 for THI, the lying time was in general lower than previous classes (Figure 9), ranging from 9 to 10.5 h with a peak of 11 h in a sunny and windy day. Also, in this case (THI > 70), it is possible to see how the combination of THI, RAD, WIND and the presence of rain affect the expected behavior of cows.

The correlation coefficient of 0.92 (*p* < 0.001) between the predicted values and the observed showed the high correspondence between the two datasets.

## 4. Discussion

The explanatory model developed in this study through the GLM procedure highlights the importance of climatic conditions on dairy cow behavior. Indeed, GLM results and the r^2^ value (0.84) represent the quota of data variability explained by the model. The high significance of the interaction of THI, RAD, RAIN and WIND, make evident the strong impact of all these factors on dairy cows’ lying behavior. Although it is already known the effect of each individual factor on cow behavior [37,38,39], there is limited knowledge on the interaction of those factors.

As it is possible to see from Figure 5, Figure 6, Figure 7, Figure 8 and Figure 9, the interaction of all those parameters has a strong and relevant effect on the lying behaviors of dairy cows. The expected lying time is quite close to values reported in literature. Indeed, the results of this study gave an average expected lying time of 9–12 h, perfectly in line with what was reported by Brzozowska et al. [40] and Herbut and Angrecka [25].

All the changes in lying behavior are the result of the combination of climatic conditions, and they should be evaluated as the expected daily lying duration. In general, climatic conditions can induce a strong thermoregulatory response in cows [41], and animal-based indicators, such as lying and feeding behaviors, provide early signs of heat stress more accurately than environmental indicators, enabling individual sensitivity to heat stress to be estimated with precision [42].

What emerges first is the fact that with low THI, low RAD, high WIND and the presence of RAIN, cows tend to stay more in the cubicle.

Longer time in the cubicle (more than 12 h) is sometimes associated with higher temperature, high WIND and high RAD, as in Figure 6 and Figure 7 (THI = 57–62 and THI = 62–69), and this could be explained by the comfort experienced by cows during the conjunction of these effects, indeed, that air movement underneath the shade is an important feature of shade to be an attractive resource to cattle [43]. Moreover, sprinklers are activated automatically when the THI exceeds the value of 68, giving relief to cows.

Furthermore, several authors found that the effect of RAD has a great impact on dairy cows, affecting how they behave in terms of barn usage and physiological changes [37,39,44]. Indeed, when RAD is too intense, cows prefer shaded areas instead of zones exposed to the direct sunlight [37,44].

As expected, high levels of THI (higher than 70) were associated to lower periods spent in the cubicle (Figure 8), and even if when the WIND increases (higher than 1.8 m/s), cows prefer to spend more time in the cubicle. Their behavior could be explained by the cooling effect resulting from the increased WIND and their preference to shaded areas [25,43].

It is quite evident that the effect of heat stress has been well mitigated by the cooling system of the barn, except for the middle of June where the THI increased suddenly, and the cows reacted to the THI change, decreasing the lying time.

In literature, since the mid-1960s, a lot has been done regarding the influence of THI on dairy cows, especially to evaluate the effects of heat stress. Lately, the THI and its effect on cows’ behavior was investigated. Indeed, modification in the lying behavior is one of the most common effects of heat stress in dairy cows [9,25,30,45]. In all the reported studies, there was a decrease of time spent lying as the THI increased, highlighting the strong relation and the high impact of this parameter on this behavior.

Regarding the effect of the rain, only in a few cases did behaviors seemed to vary from situations without it. As reported in Figure 5, Figure 6, Figure 7, Figure 8 and Figure 9, only in the case of extreme conditions, such as low THI, high WIND and low RAD, RAIN affected the behavior, decreasing the lying time (Figure 6), as reported by several studies [38,46,47].

RAIN alone cannot explain the changes in lying behavior in a roofed barn, but the combination with the other parameters could represent a discomfort situation for dairy cows [38,42,47,48].

All the effects included in the model affected significantly the lying behavior (*p* < 0.001), and this was confirmed by the high correlation coefficients between the expected and the observed values.

## 5. Conclusions

The aim of the present study was the evaluation of the interaction of climatic conditions (THI, RAD, WIND and RAIN) on the lying behavior of a group of dairy cows. The developed model seems helpful into identifying the changes in lying behavior of dairy cows according to climatic conditions. This explanatory model, if validated with the data collected in other facilities with an appropriate sample of animals, associated with the use of accelerometers, could be a valid early warning system to identify soon any deviation from the expected behavior. Potentially, this model could be also used to evaluate the goodness of barn management, and to evaluate the heat stress mitigation strategy.

## Figures and Tables

**Table 1:** List of the parameter and class division.

Parameter	Short Name	N° of Levels	Classes
THI	THI	5	THI < 50; THI = 50–57; THI = 58–62; THI = 63–69; THI > 70
Solar radiation	RAD	4	W/m^2^ < 40; W/m^2^ = 40–120; W/m^2^ = 121–240; W/m^2^ > 240
Air Velocity	WIND	4	Calm < 1 m/s; Light Air = 1.3–1.8 m/s; Light breeze = 1.3–1.8 m/s; Gentle breeze > 1.8 m/s
Precipitation	RAIN	2	No Rain = 0 mm; Rain > 0 mm
